# Higher levels of protective parenting are associated with better young adult health: exploration of mediation through epigenetic influences on pro-inflammatory processes

**DOI:** 10.3389/fpsyg.2015.00676

**Published:** 2015-05-28

**Authors:** Steven R. H. Beach, Man Kit Lei, Gene H. Brody, Meeshanthini V. Dogan, Robert A. Philibert

**Affiliations:** ^1^Center for Family Research, University of Georgia, Athens, GA, USA; ^2^Department of Biomedical Engineering, University of Iowa, Iowa City, IA, USA; ^3^Department of Psychiatry, University of Iowa, Iowa City, IA, USA

**Keywords:** epigenetic, methylation, african american, parenting, CpG, TNF, SES

## Abstract

The current investigation was designed to examine the association of parenting during late childhood and early adolescence, a time of rapid physical development, with biological propensity for inflammation. Based on life course theory, it was hypothesized that parenting during this period of rapid growth and development would be associated with biological outcomes and self-reported health assessed in young adulthood. It was expected that association of parenting with health would be mediated either by effects on methylation of a key inflammatory factor, Tumor necrosis factor (TNF), or else by association with a pro-inflammatory shift in the distribution of mononuclear blood cells. Supporting expectations, in a sample of 398 African American youth residing in rural Georgia, followed from age 11 to age 19, parenting at ages 11–13 was associated with youth reports of better health at age 19. We found that parenting was associated with changes in *TNF* methylation as well as with changes in cell-type composition. However, whereas methylation of *TNF* was a significant mediator of the association of parenting with young adult health, variation in mononuclear white blood cell types was not a significant mediator of the association of parenting with young adult health. The current research suggests the potential value of examining the health-related effects of parenting in late childhood and early adolescence. Further examination of protection against pro-inflammatory tendencies conferred by parenting appears warranted.

## Introduction

Recent theorizing has suggested that feeling sick and acting sick are adaptive elements in the response to a variety of ailments (cf. Hart, 1988; Dantzer and Kelley, 2007), and that upregulated inflammatory pathways may play an important signaling role that facilitates the perception of being sick and organizes illness-related behavior. To the extent that inflammatory mechanisms play a central role in regulating perceptions of health and illness, self-reported health potentially provides a useful window on inflammatory mechanisms. Given the importance ascribed to pro-inflammatory processes in long-term health outcomes and particularly long-term cardiovascular health (Hansson, 2005), better understanding factors contributing to pro-inflammatory tendencies has important implications for models of healthy aging. In particular, a range of social circumstances influence inflammatory processes and health, with negative social interactions resulting in greater inflammation and positive interaction leading to less inflammation (Dickerson et al., 2009; Chiang et al., 2012).

The effect of family influences during youth and early adolescence on later young adult inflammatory processes are anticipated by predictive adaptive response (PAR) models (Gluckman et al., 2005; Rickard and Lummaa, 2007), which note that if earlier family circumstances signal increased probability of future injury and/or pathogen exposure, it is potentially adaptive to prepare a developing young person to have greater inflammatory response potential (cf. Cole et al., 2011). From this perspective, social adversity and perceived threat in childhood should upregulate the innate immune system, a system that provides immediate defense against infection, and enhance pro-inflammatory response tendencies as a way to prepare for potential impending tissue damage and infection. PAR models are also consistent with broader life history theory (Charnov, 1993) in suggesting that enhanced pro-inflammatory tendencies in young adulthood may be triggered by adverse social circumstances during childhood even if such adjustments carry with them the cost of longer-term negative health implications (cf. Belsky et al., 1991; Gibbons et al., 2012).

A series of studies have identified a number of facets of parenting that may contribute to a “protective” approach to parenting in difficult circumstances that may reduce perceived threat and adversity for youth (Brody et al., 1994, 2002, 2004; Brody and Flor, 1998; Wills et al., 2000, 2003; Gibbons et al., 2004). These “protective parenting” practices foster self-regulation, academic competence, psychological adjustment, and avoidance of substance related problems (Wills et al., 2000; Brody et al., 2002; Gibbons et al., 2004) among African American youth. The cluster of protective parenting practices includes both high levels of nurturant, involved, and supportive parenting interactions as well as low levels of harsh or inconsistent parenting. Such parenting interactions lead to a lower frequency of destructive arguments and a high level of youth perceived support from parents. The constellation of “protective parenting” practices conveys security, stability, and safety at home, creating a context that would be expected to protect against pro-inflammatory remodeling. Whereas as parenting relationships characterized by lack of support and harsh parent-child interaction would be expected to have a pro-inflammatory effect.

Better understanding of the way family processes during childhood contribute to or protect against inflammation is of particular importance for African Americans who have greater prevalence, earlier onset and more complications from inflammation related diseases, including cardiovascular disease (Hozawa et al., 2007), and type 2 diabetes. African Americans also have a 30% greater chance of dying of cardiovascular disease relative to whites (Office of Minority Health, 2012), a twofold greater risk of type-II diabetes and increased likelihood of being affected by complications of type 2 diabetes, including heart disease, blindness, amputations, stroke and death (Konen et al., 1999). Increasing evidence suggests these conditions and complications are driven by inflammatory processes). African Americans also tend to show higher levels of inflammatory markers (Geronimus et al., 2006; Chyu and Upchurch, 2011; Paalani et al., 2011) than do whites. Thus, better understanding factors that protect against inflammatory processes may be particularly useful in identifying risk and protective factors for African American health in young adulthood.

Life history theory (Charnov, 1993) provides a broad framework for hypothesizing two mechanisms that may relate parenting to inflammation, particularly parenting during periods that are characterized by rapid developmental change such as late childhood and early adolescence. Less supportive and more harsh parenting should accelerate speed of development (Belsky et al., 1991; Figueredo et al., 2005) and lead to shifts in the innate immune system to better respond to a harsh interpersonal environment. Recent theorizing within this tradition suggests that these shifts may be manifested in changes in the relative frequency of particular cell types in blood (Irwin and Cole, 2011) as well as in the epigenetic programming and gene expression of such cells (Miller et al., 2011a). These two different types of effects on early programming of adaptive responses (Gluckman et al., 2005; Rickard and Lummaa, 2007) suggest the need to examine two indirect pathways through enhanced inflammatory potential to health outcomes among young adults. At the same time, epigenetic effects of parenting are plausible given previously observed epigenetic effects from parenting to later outcomes through effects on methylation in animal models (Champagne et al., 2008; Trollope et al., 2011), and a growing body of research reporting epigenetic effects of early childhood experience for humans (e.g., Essex et al., 2013; Beach et al., 2014).

If verified, the influence of protective parenting in later childhood and early adolescence on young adult health may be particularly important because parenting practices are a potentially modifiable point of intervention that could be used to ameliorate health disparities (Brody et al., 2012). Prior work has shown that family support and problem-solving skills delivered during later childhood and early adolescence can help protect youth from adverse physiological stress reactions (Chen et al., 2011; Brody et al., 2014) whereas parental maltreatment or other adverse events in childhood contribute to vulnerability to chronic diseases later in life (Repetti et al., 2002; Shonkoff et al., 2009).

Although the way in which protective parenting during childhood and adolescence can be turned into biological changes with health consequences for young adulthood is not yet fully understood (cf. Hertzman, 1999), one way that upregulated propensity for inflammation could occur is through epigenetic programming of immune cells (Miller et al., 2011a) by changing methylation of specific CpG sites (i.e., regions of DNA in which a cytosine nucleotide occurs next to a guanine nucleotide separated by only one phosphate), thereby influencing access to elements controlling rate of gene transcription. Because methylation of CpG islands associated with the first exon is particularly predictive of gene expression (e.g., Plume et al., 2012), characterizing individual differences in methylation of inflammation-related genes in the region of the first exon may be particularly informative. A second pathway from parenting to effects on inflammatory tendencies could result from changes in the relative frequency of particular immune cell types linked to inflammation (Irwin and Cole, 2011). Individuals with more cells from the innate immune system would be prepared to mount a more robust innate immune system response, providing a greater pro-inflammatory context for reactions to the environment. More specifically, epigenetic programming of cells would allow them to show more pronounced inflammatory responses when exposed to challenge (Miller et al., 2011a), an effect that has been observed in primate models (Cole et al., 2012), as well as in humans (Irwin and Cole, 2011). Likewise, a changed distribution of inflammation related cells, such as an increase in the proportion of innate immune system cells such as monocytes (aka CD14 cells) relative to T or B cells (aka CD4, CD8, and CD19) could indicate a shift toward a pro-inflammatory response pattern.

**FIGURE 1:**
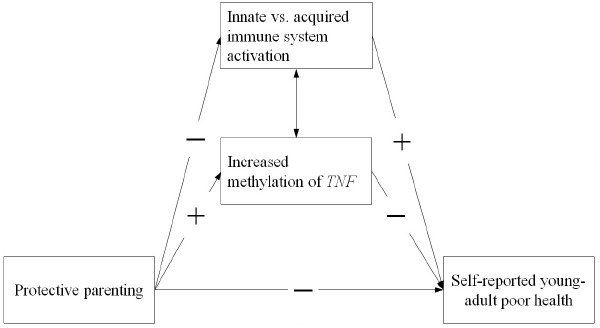
**The conceptual model showing two potential biological, indirect pathways from protective parenting to self-reported young adult poor health.** (1) A positive efffect (+) on level of methylation of TNF, and (2) a negative effect (–) on activation of monocytes reflecting greater innate immune system activation, with each associated in turn with young adult health.

The two different types of potential effects on early programming of pro-inflammatory responses (Gluckman et al., 2005; Rickard and Lummaa, 2007) suggest the need to examine two indirect pathways in models examining potential biological mechanisms of influence from parenting to later health outcomes. With regard to epigenetic change of inflammation related genes, we focus on methylation of *TNF* due to the central role of TNF-α in inflammatory processes. All cells involved in inflammation have receptors for TNF-α and are activated by it to facilitate further inflammation. This positive feedback quickly amplifies the acute phase inflammatory response, making *TNF* an attractive focus of empirical attention. With regard to cell-type variation, we examine variation in concentration of cell-types in lymphocyte pellets to identify variation that may reflect relatively greater responsiveness of the innate vs. the acquired immune system. The resulting general model is portrayed in Figure 1. To the extent that the significant indirect effects (IE) suggested in the figure are identified, they focus theoretical attention on biological mechanisms potentially conferring long-lasting effects of protective parenting on health. Accordingly, we examine the hypothesis that protective parenting during childhood and early adolescence will be associated with self-reported health in young adulthood, leading to a negative (-) association and that inflammatory mechanisms in the form of differences in *TNF* methylation and cell-type variation will account for some or all of this association. In the process of examining three main hypotheses, we also examine three measurement hypotheses:

Main hypotheses

1.
  (a) Protective parenting will be associated with epigenetic regulation of inflammation via methylation of *TNF* resulting in a positive (+) association as well as with relative activation of innate vs. acquired immune responses via shifts in the relative proportion of different white blood cell types of different white blood cell types leading to a negative (-) association.
  (b) Protective parenting will be associated with perceived health, indicating its implications for predicting young adult health.
2.
  (a) The methylation index created to capture variation in level of methylation of exon 1 of *TNF*, and a cell-type index capturing a shift toward pro-inflammatory processes, will demonstrate associations with level of protective parenting as well as with perceived health, allowing direct comparison of their role in mediating the impact of protective parenting on young adult health.
  (b) Level of methyation of *TNF* will be associated with cell-type variation such that hypothesized pro-inflammatory patterns will tend to be mutually reinforcing.
3. As portrayed in Figure 1, both methylation of *TNF* and shifts in cell-type variation will mediate the effect of protective parenting on perceived health in young adulthood and be associated with significant IE from parenting to health.

Measurement Hypotheses:

1. Batch and Chip effects will be identified in the full set of CpGs assessed and confirmed using technical replicates, allowing them to be controlled.
2. Cell type variation will be characterized utilizing patterns of DMRs, allowing characterization of shifts in relative proportions of cells associated with innate vs. acquired immune system response.
3. Degree of methylation of CpG residues on the first exon of *TNF* will be intercorrelated, indicating coordination of methylation across the first exon, and indicating that an index of methylation of the first exon for *TNF* results in a reliable construct.

## Materials and Methods

### Participants

A total of 398 primary caregivers (PCs) and target youth residing in rural Georgia, were selected randomly from a larger sample of youth participating in an ongoing longitudinal study, and provided data yearly between youth ages of 11 and 19. Participants resided in nine rural counties in Georgia. In these counties, families live in small towns and communities in which poverty rates are among the highest in the U.S. and unemployment rates are above the national average (Dalaker, 2001). Recruitment and data collection procedures were developed with input from focus groups of rural African American community members (Kumpfer et al., 2002; Murry and Brody, 2004), and community liaisons were used to aid in the recruitment and retention of participants. Community liaisons were African American community members selected on the basis of their social contacts and standing in the community. The community liaisons sent a letter to the families and followed up on the letter with phone calls to the PCs. To enhance rapport and cultural understanding, African American students and community members served as home visitors to collect data at all visits.

Self-report questionnaires were administered to mothers and target children in an interview format. Each interview was conducted privately, with no other family members present or able to overhear the conversation. Assessments of parenting were provided by African American PCs. 96.2% of PCs were female, with 89.21% being the biological mother. Other females in the role of PC were Aunt (1.05%), Grandmother 4.21%, step-mother (0.26%). When males were the PCs they were the biological father (3.42%). Foster father (0.26%), or grandfather (0.79%). Mean age of PCs was 46.122 (SD = 7.540), 15.70% had less than a 12th grade education, and 65.50% had a job. At the first assessment, PCs in the sample worked an average of 39.9 h per week, and 42.3% lived below federal poverty standards, with a majority living below 150% of the poverty threshold, and median monthly family income was $1,710. Median monthly family income was $1,648 at the age of 19. In this and other regards they are representative of the Georgia counties in which they reside (Boatright and Bachtel, 2003).

Youth provided blood for epigenetic assessments as well as reports of the caregiver’s parenting and their own physical health. Target youth mean age was 11.7 years at the first assessment and 19.2 years at the time of epigenetic assessment based on a blood draw. Of the 398 targets, 0.8% were married at the time of the blood draw, and another 1.5% were separated, divorced, or widowed. Of the young adults whose outcomes are the focus of the investigation, 45.4% are males and 54.6% of females. Approximately one-quarter (24%) had less than a 12th grade education, and only 7.3% had a full-time job.

The current sample has been the focus of prior research described in Beach et al. (in press), and Beach et al. (2014).

### Procedure

A standardized assessment protocol lasting 2 h, on average, was used at each wave of data collection. All data were collected in participants’ residences using two African American field researchers who met with each family to allow separate and simultaneous data collection from the PC and the target youth. All interviews were conducted so that no other family members were present or able to overhear the conversation. PCs consented to their own and the youths’ participation in the study, and the youths under 18 assented to their own participation and then consented when they participated as adults. All procedures were approved by the University of Georgia Institutional Review Board.

### Measures

#### Parenting

Protective parenting processes related to support, communication, and monitoring as well as adverse practices such as harsh parenting were assessed across five scales using target youth reports as well as parent reports. Three scales were common to youths and PCs with wording changes as appropriate. The Interaction Behavior Questionnaire (IBQ; Prinz et al., 1979), Nurturant-Involved Parenting Scale (Conger et al., 1994), and the Harsh/Inconsistent Parenting Scale (Brody et al., 2001) were completed by both youths and a PC. Youths also completed a revised version of the four-item Social Support for Emotional Reasons subscale from the COPE scale to assess levels of parental support (Carver et al., 1989). PCs also completed the Destructive Arguing Inventory to assess styles of conflict and conflict resolution within parent-child relationship (Kurdek, 1994). Not including harsh parenting, Cronbach’s alphas ranged from 0.73 to 0.84 for caregivers and from 0.76 to 0.85 for youth. Harsh parenting displayed lower alphas than other measures (0.54 to 0.60 for caregivers; 0.53 to 0.59 for youths).

Each scale was standardized and then averaged across the first three waves of assessment (i.e., ages 11–13). We reversed negatively valenced parenting measures to ensure that for all measures, higher scores indicated more protective parenting and fewer negative practices. All the parenting measures were summed to form the overall measure of protective parenting.

#### Young Adult Health

Youths reported their general health in young adulthood (age 19) using the General Health Perceptions subscale from the RAND Short-Form Health Survey (Hays et al., 1993) shortly after the blood draw to assess methylation. This five-item subscale includes a single-item rating of overall health ranging from 1 (*excellent*) to 5 (*poor*) and four items assessing youths’ ratings of their current health status ranging from 1 (*definitely false*) to 5 (*definitely true*); e.g., “I am as healthy as anybody I know”; “I seem to get sick a little easier than other people.” In keeping with standard scoring, responses 1 through 5 were recoded to values of 100, 75, 50, 25, 0. Positive items were reversed scored so that higher scores indicated more health problems and poorer general health. After reverse scoring, all items were averaged to yield a General Health Problems score with a range of 0–100 (α = 0.76).

#### BMI and Diet

Body mass index (BMI) was calculated at ages 18 and 19 as weight in kilograms divided by the square of height in meters. In the current study, mean BMI was 28.33 (SD = 8.13), with 54.6% of the participants classified as overweight (BMI ≥ 25). Healthy diet was assessed at ages 18 and 19 using two items that asked about frequency of fruit and vegetable consumption during the previous 7 days. The relationship between the two items was significant r = 0.483 (*p* < 0.001). Responses ranged from 1 (none) to 5 (twice a day or more) and were averaged to form the healthy diet variable.

#### Methylation

Certified phlebotomists drew four tubes of blood (30 ml) from each participant. Tubes were shipped the same day to a laboratory for preparation. After receipt, the blood tubes were inspected to ensure anticoagulation and aliquots of blood were diluted 1:1 with phosphate buffered saline pH 8.0. Mononuclear cell pellets were separated from the diluted blood specimen by centrifugation with ficoll (400 g, 30 min) and the mononuclear cell layer was removed from the tube using a transfer pipette, resuspended in a phosphate buffered saline solution, and briefly centrifuged again. The resulting cell pellet was resuspended in a 10% DMSO/RPMI solution and frozen at –80°C until use. A typical DNA yield for each pellet was between 10 and 15 mg.

The Illumina (San Diego, CA, USA) HumanMethylation450 Beadchip was used to assess genome-wide DNA methylation. Participants were randomly assigned to 12 sample “slides/chips” with groups of eight slides being bisulfite converted in a single batch, resulting in five “batches/plates.” A replicated sample of DNA was included in each plate to aid in assessment of batch variation and to ensure correct handling of specimens. The replicate sample was examined for average correlation of beta values between plates and was found to be greater than 0.99. Prior to normalization, methylation data were filtered based on these criteria: (1) samples containing 1% of CpG sites with detection p-value > 0.05 were removed, (2) sites were removed if a beadcount of < 3 was present in 5% of samples and (3) sites with a detection *p*-value of > 0.05 in 1% of samples were removed. More than 99.76% of the 485,577 probes yielded statistically reliable data.

***Quantile normalization of methylation data***

Recent demonstrations (e.g. Pidsley et al., 2013) have shown that quantile normalization methods as well as separate normalization of Type I and Type II assays in the Illumina array produce marked improvement in detection of relationships by correcting distributional problems inherent in the manufacturers default method for calculating β (i.e., β = *M*/(*M* + *U* + 100) where M and U are methylated and unmethylated signal intensities, respectively. Accordingly, for the current investigation, the methylated and unmethylated intensities obtained using the Illumina Human-Methylation450 BeadChip were quantile normalized using the wateRmelon (2013) R package (Team, 2012; Schalkwyk et al., 2013). The “*dasen”* function recommended by Pidsley et al. (2013) was used. This method equalizes the backgrounds of Type I and Type II probes prior to normalization, and includes between-array normalization of Type I and Type II probes separately but does not perform dye bias correction.

***Identifying and correcting for chip and batch effects***

As demonstrated by Sun et al. (2011), quantile normalization typically reduces, but may not eliminate, batch and chip effects. Accordingly, after preprocessing to normalize the beta values, all samples were examined for batch and chip effects. The distribution of quantile normalized average β values for all samples in each chip and batch were contrasted with all others using a box and density plot to indicate both the mean and confidence intervals around the mean in each case. The results of this examination are provided in the preliminary stage of the results section. Any plate or chip effects can then be controlled in all subsequent analyses. Because all plates contained a technical replicate, it was also possible to confirm the batch/plate effects via direct examination of the replicated sample.

***Assessing proportion of cell types in mixed cell populations***

Ficoll purified peripheral blood mononuclear cell pellets of the sort used in the current investigation are comprised of several different cell types (e.g., CD4, CD8, CD14, CD19, CD56) (Reinius et al., 2012). To account for individual differences in cell types, such as that potentially produced by a shift toward pro-inflammatory innate immune system cell types, a regression calibration approach similar to that developed by Houseman et al. (2012) was performed. However, whereas Houseman et al. (2012) used an approach based on the Illumina HumanMethylation 27K BeadChip, we utilized an alternative approach based on Illumina HumanMethylation 450K BeadChip data. Highly informative CpG sites were identified by contrasting methylation profiles for purified cells (CD4+ T cells, CD8+ T cells, CD14+ monocytes, CD19+ B cells, and CD56+ Natural Killer cells) using data contributed by (Reinius et al., 2012; GEO database under accession number GSE35069) and analyses were performed using regression in MethLAB, Version 1.5 (Kilaru et al., 2012). The 100 sites best differentiating to the five cell types of interest were retained for further analysis in SPSS version 22 (IBM Corporation Released, 2013). A locus determined to be on the X chromosome was dropped from subsequent analyses. Then, we performed a principal components analysis (PCA) on the remaining 99 loci using the current data set to identify principle component factors that would characterize dimensions of individual variability in cell-type in the most parsimonious manner for the current data set.

## Results

Results are presented in six steps, beginning with the three steps reflecting preliminary, measurement-related analyses and then the three steps related to specific hypotheses.

### Measurement-related Analyses

#### Characterizing Cell Type Variation

As described above, we extracted the 99 loci best differentiating cell-types in the Reinius et al. (2012) data set, and then subjected these to a principle component factor analysis. The scree plot had an elbow after the first three factors, with the first three factors accounting for 31.06%; 14.26%; and 6.64% of the common variance respectively. To determine which, if any of these factors might reflect a shift toward greater innate vs acquired immune response, we used a simultaneous regression to predict factor scores from cell types using the original data set from Reinius et al. (2012). Greater scores on the first principle component (PC1) were inversely associated with CD56 (Natural Killer Cells) and positively associated with CD4 (T helper cells) and CD19 (B or T helper cells), suggesting a relationship to both innate and acquired immune system activity. The second principle component (PC2) was associated negatively with CD19 (B or T cells) and positively with CD8 (T suppressor cells), albeit non-significantly in all cases, a pattern not clearly reflective of a shift toward innate or acquired responses. PC3 was positively and robustly associated with CD14 (monocytes) and negatively associated with CD19 (acquired B or T cells), suggesting a shift toward greater responsiveness of the innate immune system. All principle component scores were also examined at the zero order level to identify associations with parenting and young adult health to identify which could potentially mediate effects of protective parenting.

#### Characterizing Batch and Chip Effects

**FIGURE 2:**
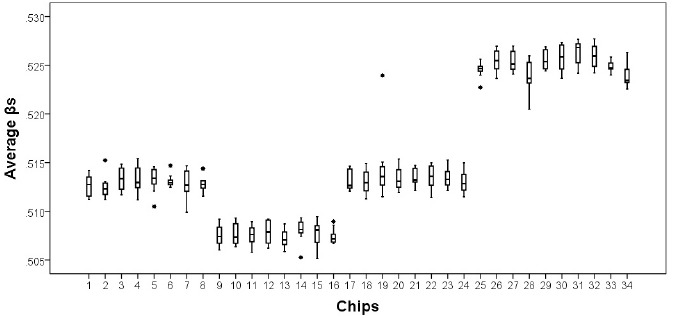
**Average methylation values across all CpG sites by chip.** Chips 1–8 chips on Plate 1; chips 9–16 chips on Plate 2; chips 17–24 on Plate 3; chips 25–32 on Plate 4; chips 33–34 on Plate 5. There are plate effects but not chip effects, with Plates 4 and 5 having higher average methylation values and Plate 2 having a lower average Beta than do Plates 1 and 3. *indicates the presence of an outlier from the chip.

To examine the distribution of quantile normalized average βs indicating level of methylation, all methylation values for each sample were contrasted with all others using a box and density plot to indicate both the mean and confidence intervals around the mean for each chip and batch. As can be seen in Figure 2, confidence intervals for chips within batches overlap suggesting no need for correction of chip effects. However, confidence intervals for batches did not overlap, suggesting there is a need for correction of batch/plate effects.

The pattern of means observed for the technical replicate reproduced the pattern observed across all quantile normalized average βs, confirming the need to correct for batch/plate effects. Accordingly, batch/plate was used as a categorical covariate in all analyses.

#### TNF Methylation

Eight CpG sites were identified as being associated with the first exon of *TNF*. Greater methylation of this region for cells expressing TNF should result in a reduction in gene expression and so, ultimately, a reduction in TNF-α. The inter-correlation of the eight CpG values were examined (*r*s ranging from 0.736 to 0.942; all *p*s < 0.00001) and all individual CpGs were significantly correlated with parenting (*r*s ranging from 0.094 to 0.172) and with young adult health (*r*s ranging from –0.080 to –0.143). A factor analysis of the eight CpGs identified a single factor with all loadings above 0.85. Accordingly, to index overall methylation of the first exon of *TNF*, βs for CpGs on the first exon were mean-centered and standardized prior to creating an average score with a Cronbach alpha of 0.98.

### Model-related Analyses

#### Hypothesis 1a

As can be seen in Table 1, protective parenting was associated with the *TNF*-index (*r* = 0.150, *p* = 0.003). However, of the three principle components comprising variation in cell types, protective parenting was associated significantly only with PC3 (*r* = –0.166, *p* = 0.001).

**TABLE 1:** **Correlation matrix for the major study variables (*N* = 398)**.

****	**1**	**2**	**3**	**4**	**5**	**6**	**7**	**8**	**9**	**10**
1. Parenting	–								
2. Self-reported health	–0.108*	–							
3. *TNF*-index	0.150**	–0.123*	–						
4. Male	0.007	–0.095^†^	0.066	–					
5. Age	0.037	–0.124*	0.104*	0.014	–				
6. BMI	–0.005	0.098^†^	–0.034	–0.128*	0.055	–			
7. Diet	0.011	–0.079	–0.035	–0.003	0.042	0.036	–		
8. Factor 1	0.075	–0.041	0.662**	–0.149**	0.103*	0.006	–0.008	–	
9. Factor 2	0.035	–0.094^†^	0.334**	0.128*	0.093^†^	–0.008	–0.085	0.003	–
10. Factor 3	–0.166**	0.039	–0.350**	–0.218**	–0.019	0.021	0.018	–0.005	–0.011	–

Mean	–0.131	26.043	0.001	0.452	20.464	0.548	2.835	0.084	0.017	0.031
*SD*	4.375	18.721	0.938	–	0.607	0.498	0.854	30.618	14.263	6.499

**p ≤ 0.01; *p ≤ 0.05; ^†^p < 0.10 (two-tailed tests). Factors 1–3 are the three principle components reflecting cell-type variation in the current data.

#### Hypothesis 1b

Protective parenting was also associated significantly with young adults’ reports of health in young adulthood (*r* = –0.108, *p* = 0.031).

#### Hypothesis 2a

As can be seen in Table 1, the methylation index for *TNF* was associated with young adult health (*r* = –0.123, *p* = 0.014), as well as with cell-type variation (PC1 *r* = 0.662, *p* = 0.000; PC2 *r* = 0.334, *p* = 0.000; PC3 *r* = –0.350, *p* = 0.000), setting the stage for tests of mediation. However, of the factors capturing cell-type variation, only factor 3 was associated both with parenting (*r* = –0.166, *p* = 0.001). Accordingly, the *TNF*-index and Factor 3 were examined as potential alternative pro-inflammatory mediators of the effect of parenting on young adult health.

#### Hypothesis 2b

The *TNF*-index was also associated in the expected direction with PC3 (*r* = –0.350, *p* = 0.000), indicating that less methylation of *TNF* (pro-inflammatory) was associated with relatively greater presence of monocytes (pro-inflammatory).

#### Hypothesis 3

Using the *TNF*-index and PC3 to represent alternative potential pro-inflammatory pathways linking protective parenting to health, we examined whether the effects of protective parenting on self-reported health were mediated by methylation and/or cell type variation. We used the function (MODEL INDIRECT) in Mplus version 7.2 (Muthén and Muthén, 1998–2012) and obtained bootstrap confidence intervals for the effect of the independent variables (parenting and SES risk exposure) on the outcome variable (young adult self-reported health) through the mediator (methylation index) using 1000 replicates to assess the bias-corrected 95% confidence intervals for the IE (Hayes, 2009). This approach estimates direct and IE simultaneously, does not assume a standard normal distribution when calculating the *p*-value for the IE, and repeatedly samples the data to estimate the IE.

**FIGURE 3:**
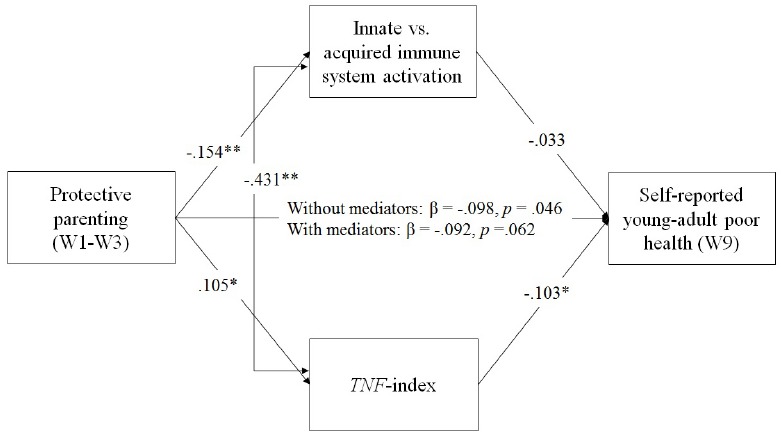
**Mediated effect of protective parenting on youth reported health in young adulthood, controlling for sex, age, and batch effects, freeing non-significant pathways from control variables.** Chi-square = 4.124, *df* = 3, *p* = 0.248; CFI = 0.996; RMSEA = 0.031. Values are standardized parameter values. Sex, age, BMI, diet, and batch/plate are controlled. *N* = 398. *p < 0.05, **p < 0.01, two-tailed.

In the final model, shown in Figure 3, which drops non-significant pathways from control variables, parenting is associated with both youth reported health and with *TNF*-methylation. We found that parenting was associated with the *TNF*-methylation index score as well as with Factor 3 reflecting cell-type variation in a multivariate context (See Figure 3), controlling for batch/plate effects. As can be seen in Table 2, the impact of protective parenting on self-reported health in young adulthood was partially, but not fully, mediated by impact on the *TNF*-methylation index, accounting for 11.19% of the variance in young adult health, with a significant IE of parenting on young adult health of *IE* = –0.047, 95% CI = (–0.127, –0.003) and a significant unstandardized direct effect of *B* = –0.42, 95% CI = (–0.790, –0.048). Factor 3, however, id not mediate the effects of parenting on young adult health, *IE* = 0.022, 95% CI = (–0.047, 0.109).

**TABLE 2:** **Significance of the indirect effects on health through factor 3 and TNF (*N* = 398)**.

**Paths**	**Total effect**	**Indirect effect**	**The portion of the variance for mediator**
Protective Parenting (W1–W3)→ Innate vs. acquired immune system activation→ Self-reported young-adult poor health (W9)	–0.420* (–0.790, –0.048)	0.022 (–0.047, 0.109)	—%
Protective Parenting (W1–W3)→ TNF-index activation→ Self-reported young-adult poor health (W9)	–0.420* (–0.790, –0.048)	–0.047* (–0.127, –0.003)	11.190%

The values presented are standardized parameters. Bootstrapping with 1,000 replications. *p ≤ 0.05, (two-tailed test).

## Discussion

The examination of the way that protective parenting during late childhood and early adolescence influences health and potentially remodels biological systems through epigenetic change is just beginning. To the best of our knowledge, this is the first study to contrast potential effects of protective parenting during late childhood and early adolescence on pro-inflammatory process through epigenetic regulation of gene expression vs. increased presence of pro-inflammatory innate immune system cell-types (e.g., monocytes). Consistent with study hypotheses, we found that protective parenting practices, assessed longitudinally, were associated with effects on pro-inflammatory processes both through effects on methylation of *TNF*, and through effects on proportion of immune cells that were monocytes. Accordingly, parenting at ages 11–13 was negatively associated with both pro-inflammatory epigenetic patterns and pro-inflammatory cell-type variation assessed at age 19. In addition, these effects were not spurious associations due to age, gender, or batch (i.e., measurement) effects. Because we used a longitudinal research design to test hypotheses regarding the effects of parenting on health, we identified protective parenting as a predictor of pro-inflammatory processes and so strengthened its claim to play a causal role in young adult health. These findings are consistent with the proposition that poor health and health disparities during young adulthood may be ameliorated, in part, by processes correlated with protective parenting.

The current results suggest that better understanding the way in which mononuclear white blood cell signaling processes are altered, net of effects on individual differences in cell type composition, is promising as a mechanism by which protective parenting influences young adult pro-inflammatory tendencies among African American youth. The current study builds on past results by suggesting that there may be effects of protective parenting in late childhood and early adolescence that parallel or expand upon those observed for early childhood life stress on subsequent biological and genomic functioning (e.g., Miller et al., 2011b; Essex et al., 2013). The current results suggest that protective parenting measured during late childhood and early adolescence may also exert an influence on genomic functioning and health in young adults, and contributes to promising work on multiple fronts suggesting that various epigenetic mechanisms may be related to, and help account for, long-term effects of protective parenting on health.

At the same time, our findings provided only partial support for the dual pathway model outlined in Figure 1. First, although it was possible to characterize the relative proportion of mononuclear white blood cell types in the blood samples provided using the techniques developed by Accomando et al. (2014) and Houseman et al. (2012) to characterize cell type variation, and we found that protective parenting was associated with the relative proportion of monocytes in the mononuclear white blood cell sample, that proportion was not associated with youth reports of their health in young adulthood. Accordingly, there was no evidence of mediation of parenting effects on young adult health through cell-type variation in the current sample. In addition, even the significant mediational pathway through *TNF* methylation did not account for the majority of health related effects of protective parenting during late childhood and early adolescence. Only 11.19% of the variance in young adult health attributable to protective parenting was accounted for by *TNF* methylation, suggesting that there are other pathways to young adult health that require explication.

In addition, limitations of the present study design that preclude strong causal conclusions should be noted. Because we did not assess early childhood parenting, we do not know if the observed effects for protective parenting during late childhood and early adolescence might be accounted for by even more potent effects in early childhood. Future work with measures of both early and later parenting would be helpful in resolving this concern. In addition, there were no repeated measurements of either level of *TNF* methylation or variability in cell-type composition. As a consequence, it is not possible to address the issue of change in inflammatory mechanisms and whether protective parenting in late childhood was associated with this change. It would be useful if future replications with younger rural African American children could examine the interplay of environmental challenges and parenting occurring at multiple stages of development to better characterize key developmental stages at which protective parenting exerts its greatest effects. Likewise it would be useful to examine whether protective parenting has different effects on inflammatory processes at different ages. Accordingly, replication of the current investigation with repeated measurement of methylation as well as repeated measurement of protective parenting would be useful. Finally, because we did not measure gene expression, the current results await replication and extension using methods that can clarify whether the observed effects are associated with changes in gene expression that would indicate up or down regulation of *TNF*. Additional limitations include absence of objective medical records, lack of information about possible in utero exposures, lack of specific somatometric assessments of truncal fat, lack of specific dietary assessment of PUFA consumption, lack of explication of potential co-caregiver effects, and absence of genotyping of TNF polymorphisms. However, we controlled for BMI and general aspects of healthy diet and found no significant association with TNF methylation, finding that these did not affect the observed relationships. Likewise, self-reported health just prior to the blood draw indicated that the young adults who comprised the current sample considered themselves relatively healthy on average at the time of the blood draw and they were not yet at an age where a general population sample of this sort would be anticipated to have accumulated large numbers of physician documented health problems. Finally, to the extent that specific genetic polymorphisms influence TNF methylation, they would not be anticipated to affect the observed results unless they were correlated with parenting as well as TNF methylation.

There is also room for more fine-grained examination of parenting. Protective parenting is comprised of both increased positive and decreased negative forms of parent-child interaction. Accordingly, more fine-grained examination of different facets of parenting could be useful in determining whether some facets of parenting are particularly consequential with regard to particular outcomes. More fine grained analyses could be useful, for example, in developing implications for more targeted intervention to enhance health. There is also a particular need for replication and extension of our finding that greater protective parenting was associated with a significant shift in cell-type composition in the direction of relatively fewer CD14 monocytes (a marker of innate immunity), suggesting potential long-term impact on chronic inflammation (Irwin and Cole, 2011). Although controlling the effect of cell type did not reduce the direct effect of parenting on health or result in a significant IE of protective parenting on young-adult health, this effect does illustrate the potential for protective parenting to influence future health-relevant processes indirectly by changing cell type. To the extent that such changes in cell-type composition enhance reactivity to future life stress, exacerbate the negative effects of health behaviors, accelerate other aging related processes, or change signaling processes that influence other behavior patterns, they may be quite consequential for long-term health outcomes even though that is not apparent in the current investigation. Accordingly, future examination of effects related to impact on cell-type variation is warranted.

Despite limitations and the need for future replication, the current results provide a useful demonstration of the impact of protective parenting during pre-adolescence on *TNF* methylation and youths’ long-term health outcomes. Jointly, the results suggest the value of continued investigation of epigenetic changes related to protective parenting and its potential for impact on later health outcomes.

### Conflict of Interest Statement

The authors declare that the research was conducted in the absence of any commercial or financial relationships that could be construed as a potential conflict of interest.

## Abbreviations

CpG: regions of DNA in which a cytosine nucleotide occurs next to a guanine nucleotide separated by only one phosphate
TNF: Tumor necrosis factor
SES: socioeconomic status
DMR: Differentially methylated Region

## References

[B1] Accomando W. P. Wiencke J. K. Houseman E. A. Nelson H. N. Kelsey K. T. (2014). Quantitative reconstruction of leukocyte subsets using DNA methylation. Genome Biol. 15:R50. 10.1186/gb-2014-15-3-r50 24598480 PMC4053693

[B2] Beach S. R. H. Brody G. H. Lei M. K. Kim S. Cui J. Philibert R. A. (2014). Is serotonin transporter genotype associated with epigenetic susceptibility or vulnerability? Examination of the impact of socioeconomic status risk on African American youth. Dev. Psychopathol. 26, 289–304. 10.1017/S0954579413000990 24438855 PMC4118288

[B3] Beach S. R. H. Lei M. K. Brody G. H. Kim S. Barton A. W. Dogan M. V. (in press). Parenting, SES-risk, and later Young Adult Health: exploration of opposing indirect effects via DNA methylation. Child Dev. 10.1111/cdev.12486 PMC4733891 26822447

[B4] Belsky J. Steinberg L. Draper P. (1991). Childhood experience, interpersonal development, and reproductive strategy: and evolutionary theory of socialization. Child Dev. 62, 647–670. 1935336 10.1111/j.1467-8624.1991.tb01558.x

[B5] Boatright S. R. Bachtel D. C. (2003). The Georgia County Guide, 19th Edn. Athens, GA: University of Georgia Cooperative Extension Service.

[B6] Brody G. H. Chen Y.-F. Kogan S. M. Yu T. Molgaard V. K. DiClemente R. J. (2012). Family-centered program to prevent substance use, conduct problems, and depressive symptoms in Black adolescents. Pediatrics 129, 108–115. 10.1542/peds.2011-0623 22157131 PMC3255466

[B7] Brody G. H. Flor D. L. (1998). Maternal resources, parenting practices, and child competence in rural, single-parent African American families. Child Dev. 69, 803–816. 10.1111/j.1467-8624.1998.tb06244.x 9680686

[B8] Brody G. H. Ge X. Conger R. D. Gibbons F. X. Murry V. M. Gerrard M. (2001). The influence of neighborhood disadvantage, collective socialization, and parenting on African American children’s affiliation with deviant peers. Child Dev. 72, 1231–1246. 10.1111/1467-8624.00344 11480944

[B9] Brody G. H. Murry V. M. Gerrard M. Gibbons F. X. Molgaard V. McNair L. (2004). The strong african american families program: translating research into prevention programming. Child Dev. 75, 900–917. 10.1111/j.1467-8624.2004.00713.x 15144493

[B10] Brody G. H. Murry V. M. Kim S. Brown A. C. (2002). Longitudinal pathways to competence and psychological adjustment among African American children living in rural single-parent households. Child Dev. 73, 1505–1516. 10.1111/1467-8624.00486 12361315

[B11] Brody G. H. Stoneman Z. Flor D. McCrary C. Hastings L. Conyers O. (1994). Financial resources, parent psychological functioning, parent co-caregiving, and early adolescent competence in rural two-parent African-American families. Child Dev. 65, 590–605. 10.2307/1131403 8013241

[B12] Brody G. H. Yu T. Beach S. R. H. Kogan S. M. Windle M. Philibert R. A. (2014). Harsh parenting and adolescent health: a longitudinal analysis with genetic moderation. Health Psychol. 33, 401–409. 10.1037/a0032686 23668852 PMC4534086

[B13] Carver C. S. Scheier M. F. Weintraub J. K. (1989). Assessing coping strategies: a theoretically based approach. J. Pers. Soc. Psychol. 56, 267–283. 10.1037/0022-3514.56.2.267 2926629

[B14] Champagne D. L. Bagot R. C. van Hasselt F. Ramakers G. Meaney M. J. de Kloet E. R. (2008). Maternal care and hippocampal plasticity: evidence for experience-dependent structural plasticity, altered synaptic functioning, and differential responsiveness to glucocorticoids and stress. J. Neurosci. 4, 28, 6037–6045. 10.1523/JNEUROSCI.0526-08.2008 18524909 PMC6670331

[B15] Charnov E. L. (1993). Life History Invariants. Oxford: Oxford University Press.

[B16] Chen E. Miller G. E. Kobor M. S. Cole S. W. (2011). Maternal warmth buffers the effects of low early-life socioeconomic status on pro-inflammatory signaling in adulthood. Mol. Psychiatry 16, 729–737. 10.1038/mp.2010.53 20479762 PMC2925055

[B17] Chiang J. Eisenberger N. I. Seeman T. E. Taylor S. E. (2012). Negative and competitive social interactions are related to heightened proinflammatory cytokine activity. Proc. Natl. Acad. Sci. 109, 1878–1882. 10.1073/pnas.1120972109 22308464 PMC3277534

[B18] Chyu L. Upchurch D. M. (2011). Racial and ethnic patterns of allostatic load among adult women in the united states: findings from the national health and nutrition examination survey 1999–2004. J. Womens Health 20, 575–583. 10.1089/jwh.2010.2170 21428732 PMC3075046

[B19] Cole S. W. Conti G. Arevalo J. M. G. Ruggiero A. M. Heckman J. J. Suomi S. J. (2012). Transcriptional modulation of the developing immune system by early life social adversity. Proc. Natl. Acad. Sci. U.S.A. 109, 20578–20583. 10.1073/pnas.1218253109 23184974 PMC3528538

[B20] Cole S. W. Hawkley L. C. Arevalo J. M. G. Cacioppo J. T. (2011). Transcript origin analysis identifies antigen presenting cells as primary targets of socially regulated gene expression in leukocytes. Proc. Natl. Acad. Sci. U.S.A. 108, 3080–3085. 10.1073/pnas.1014218108 21300872 PMC3041107

[B21] Conger R. D. Ge X. Elder G. H. Lorenz F. O. Simons R. L. (1994). Economic stress, coercive family process, and developmental problems of adolescents. Child Dev. 65, 541–561. 8013239

[B22] Dalaker J. (2001). Poverty in the United States, 2000 (U. S. Census Bureau Current Population Reports Series P 60–214). Washington, DC: U. S. Government Printing Office.

[B23] Dantzer R. Kelley K. W. (2007). Twenty years of research on cytokine-induced sickness behavior. Brain Behav. Immun. 21, 153–160. 10.1016/j.bbi.2006.09.006 17088043 PMC1850954

[B24] Dickerson S. S. Gable S. L. Irwin M. R. Aziz N. Kemeny M. E. (2009). Social-evaluative threat and proinflammatory cytokine regulation: an experimental laboratory investigation. Psychol. Sci. 20, 1237–1244. 10.1111/j.1467-9280.2009.02437.x 19754527 PMC2761517

[B25] Figueredo A. J. Vásquez G. Brumbach B. H. Sefcek J. A. Kirsner B. R. Jacobs W. J. (2005). The K factor: individual differences in life history strategy. Pers. Individ. Differ. 39, 1349–1360. 10.1016/j.paid.2005.06.009

[B26] Essex M. J. Thomas Boyce W. Hertzman C. Lam L. L. Armstrong J. M. Neumann S. (2013). Epigenetic vestiges of early developmental adversity: childhood stress exposure and DNA methylation in adolescence. Child Dev. 84, 58–75. 10.1111/j.1467-8624.2011.01641.x 21883162 PMC3235257

[B27] Geronimus A. T. Hicken M. Keene D. Bound J. (2006). “Weathering” and age patterns of allostatic load scores among blacks and whites in the United States. Am. J. Public Health 96, 826–833. 10.2105/ajph.2004.060749 16380565 PMC1470581

[B28] Gibbons F. X. Gerrard M. Cleveland M. J. Wills T. A. Brody G. (2004). Perceived discrimination and substance use in African American parents and their children: a panel study. J. Pers. Soc. Psychol. 86, 517–529. 10.1037/0022-3514.86.4.517 15053703

[B29] Gibbons F. X. Roberts M. E. Gerrard M. Li Z. Beach S. R. H. Simons R. L. (2012). The impact of stress on the life history strategies of African American adolescents: cognitions, genetic moderation, and the role of discrimination. Dev. Psychol. 48, 722–739. 10.1037/a0026599 22251000 PMC4324554

[B30] Gluckman P. D. Hanson M. A. Spencer H. G. (2005). Predictive adaptive responses and human evolution. Trends Ecol. Evol. 20, 527–533. 10.1016/j.tree.2005.08.001 16701430

[B31] Hansson G. K. (2005). Inflammation, atherosclerosis, and coronary artery disease. N. Engl. J. Med. 352, 1685–1695. 10.1056/NEJMra043430 15843671

[B32] Hart B. L. (1988). Biological basis of the behavior of sick animals. Neurosci. Biobehav. Rev. 12, 123–37. 10.1016/S0149-7634(88)80004-6 3050629

[B33] Hayes A. F. (2009). Beyond baron and kenny: statistical mediation analysis in the new millennium. Commu. Monogr. 76, 408–420. 10.1080/03637750903310360

[B34] Hays R. D. Sherbourne C. D. Mazel R. M. (1993). The RAND 36-item health survey 1.0. Health Econ. 2, 217–227. 10.1002/hec.4730020305 8275167

[B35] Hertzman C. (1999). The biological embedding of early experience and its effects on health in adulthood. Ann. N. Y. Acad. Sci. 896, 85–95. 10681890 10.1111/j.1749-6632.1999.tb08107.x

[B36] Houseman E. A. Accomando W. P. Koestler D. C. Christensen B. C. Marsit C. J. Nelson H. H. (2012). DNA methylation arrays as surrogate measures of cell mixture distribution. BMC Bioinform. 13:86–101. 10.1186/1471-2105-13-86 22568884 PMC3532182

[B37] Hozawa A. Folsom A. R. Sharrett A. R. Chambless L. E. (2007). Absolute and attributable risks of cardiovascular disease incidence in relation to optimal and borderline risk factors—comparison of african american with white subjects—atherosclerosis risk in communities study. Arch. Intern. Med. 167, 573–579. 10.1001/archinte.167.6.573 17389288

[B38] IBM Corporation Released. (2013). IBM SPSS Statistics for Windows, Version 22.0. Armonk, NY: IBM Corporation.

[B39] Irwin M. R. Cole S. W. (2011). Reciprocal regulation of the neural and innate immune systems. Nat. Rev. Immunol. 11, 625–632. 10.1038/nri3042 21818124 PMC3597082

[B40] Kilaru V. Barfield R. T. Schroeder J. W. Smith A. K. Conneely K. N. (2012). MethLAB: a graphical user interface package for the analysis of array-based DNA methylation data. Epigenetics 7, 225–229. 10.4161/epi.7.3.19284 22430798 PMC3335946

[B41] Konen J. C. Summerson J. H. Bell R. A. Curtis L. G. (1999). Racial differences in symptoms and complications in adults with type 2 diabetes mellitus. Ethn. Health 4, 39–49. 10.1080/13557859998182 10887461

[B42] Kumpfer K. L. Alvarado R. Smith P. Bellamy N. (2002). Cultural sensitivity and adaptation in family-based prevention interventions. Prev. Sci. 3, 241–246. 10.1023/A:1019902902119 12387558

[B43] Kurdek L. A. (1994). Areas of conflict for gay, lesbian, and heterosexual couples: what couples argue about influences relationship satisfaction. J. Marriage Fam. 56, 923–934. 10.2307/353603

[B44] Miller G. E. Chen E. Parker K. J. (2011a). Psychological stress in childhood and susceptibility to the chronic diseases of aging: moving toward a model of behavioral and biological mechanisms. Psychol. Bull. 137, 959–997. 10.1037/a0024768 21787044 PMC3202072

[B45] Miller G. E. Lachman M. E. Chen E. Gruenewald T. L. Karlamangla A. S. Seeman T. E. (2011b). Pathways to resilience: maternal nurturance as a buffer against the effects of childhood poverty on metabolic syndrome at midlife. Psychol. Sci. 22, 1591–1599. 10.1177/0956797611419170 22123777 PMC3362047

[B46] Murry V. M. Brody G. H. (2004). Partnering with community stakeholders: engaging families in basic research and the Strong African American Families prevention intervention program. J. Marital Fam. Ther. 30, 113–129. 10.1111/j.1752-0606.2004.tb01240.x 15293647

[B47] Muthén L. K. Muthén B. O. (1998–2012). Mplus User’s Guide, 7th Edn. Los Angeles, CA: Muthén & Muthén.

[B48] Office of Minority Health. (2012). Heart Disease and African Americans. Washington, DC: U.S. Department of Health and Human Services Available at: http://minorityhealth.hhs.gov/templates/content.aspx?lvl=2&lvlID=51&ID=3018

[B49] Paalani M. Lee J. W. Haddad E. Tonstad S. (2011). Determinants of inflammatory markers in a bi-ethnic population. Ethn. Dis. 21, 142–149. 21749016 PMC3427005

[B50] Pidsley R. Wong C. C. Y. Volta M. Lunnon K. Mill J. Schalkwyk L. C. (2013). A data-driven approach to preprocessing Illumina 450K methylation array data. BMC Genomics 14:293–302. 10.1186/1471-2164-14-293 23631413 PMC3769145

[B51] Plume J. M. Beach S. R. H. Brody G. H. Philibert R. A. (2012). A cross-platform genome-wide comparison of the relationship of promoter DNA methylation to gene expression. Front. Genet. 3:12. 10.3389/fgene.2012.00012 22363339 PMC3277264

[B52] Prinz R. J. Foster S. L. Kent R. N. O’Leary K. D. (1979). Multivariate assessment of conflict in distressed and nondistressed mother–adolescent dyads. J. Appl. Behav. Anal. 12, 691–700. 10.1901/jaba.1979.12-691 541311 PMC1311489

[B53] Reinius L. E. Acevedo N. Joerink M. Pershagen G. Dahlén S. E. Greco D. (2012). Differential DNA methylation in purified human blood cells: implications for cell lineage and studies on disease susceptibility. PLoS ONE 7:e41361. 10.1371/journal.pone.0041361 22848472 PMC3405143

[B54] Repetti R. L. Taylor S. E. Seeman T. E. (2002). Risky families: family social environments and the mental and physical health of offspring. Psychol. Bull. 128, 330–336. 10.1037/0033-2909.128.2.330 11931522

[B55] Rickard I. J. Lummaa V. (2007). The predictive adaptive response and metabolic syndrome: challenges for the hypothesis. Trends Endocrinol. Metabol. 18, 94–99. 10.1016/j.tem.2007.02.004 17320410

[B56] Schalkwyk L. C. Pidsley R. Wong C. C. (2013). WateRmelon: Illumina 450 methylation array normalization and metrics [computer program]. Version R package 1.5.1.2013.

[B57] Shonkoff J. P. Boyce W. T. McEwen B. S. (2009). Neuroscience, molecular biology, and the childhood roots of health disparities: building a new framework for health promotion and disease prevention. J. Am. Med. Association 301, 2252–2259. 10.1001/jama.2009.754 19491187

[B58] Sun Z. Chai H. S. Wu Y. White W. M. Donkena K. V. Garovic V. D. (2011). Batch effect correction for genome-wide methylation data with Illumina Infinium platform. BMC Med. Genomics 16:84. 10.1186/1755-8794-4-84 22171553 PMC3265417

[B59] Team R. C. (2012). R: A Language and Environment For Statistical Computing. Vienna: R Foundation for Statistical Computing.

[B60] Trollope A. F. Gutierrez-Mecinas M. Mifsud K. R. Collins A. Saunderson E. A. Reul J. M. (2011). Stress, epigenetic control of gene expression and memory formation. Exp. Neurol. 233, 3–11. 10.1016/j.expneurol.2011.03.022 21466804

[B61] Wills T. A. Gibbons F. X. Gerrard M. Brody G. H. (2000). Protection and vulnerability processes relevant for early onset of substance use: a test among African American children. Health Psychol. 19, 253–263. 10.1037/0278-6133.19.3.253 10868770

[B62] Wills T. A. Gibbons F. X. Gerrard M. Murry V. M. Brody G. H. (2003). Family communication and religiosity related to substance use and sexual behavior in early adolescence: a test for pathways through self-control and prototype perceptions. Psychol. Addict. Behav. 17, 312–323. 10.1037/0893-164X.17.4.312 14640827

